# Localized Calcium Signaling and the Control of Coupling at Cx36 Gap Junctions

**DOI:** 10.1523/ENEURO.0445-19.2020

**Published:** 2020-04-15

**Authors:** Keith B. Moore, Cheryl K. Mitchell, Ya-Ping Lin, Yuan-Hao Lee, Eyad Shihabeddin, John O’Brien

**Affiliations:** 1 Richard S. Ruiz, M.D. Department of Ophthalmology and Visual Science, McGovern Medical School, The University of Texas Health Science Center at Houston, Houston, TX 77030; 2 The MD Anderson Cancer Center UTHealth Graduate School of Biomedical Sciences, Houston, TX 77030

**Keywords:** calcium signaling, Connexin 36, electrical synapse, optical imaging, plasticity, tracer coupling

## Abstract

A variety of electrical synapses are capable of activity-dependent plasticity, including both activity-dependent potentiation and activity-dependent depression. In several types of neurons, activity-dependent electrical synapse plasticity depends on changes in the local Ca^2+^ environment. To enable study of local Ca^2+^ signaling that regulates plasticity, we developed a GCaMP Ca^2+^ biosensor fused to the electrical synapse protein Connexin 36 (Cx36). Cx36-GCaMP transfected into mammalian cell cultures formed gap junctions at cell-cell boundaries and supported Neurobiotin tracer coupling that was regulated by protein kinase A signaling in the same way as Cx36. Cx36-GCaMP gap junctions robustly reported local Ca^2+^ increases in response to addition of a Ca^2+^ ionophore with increases in fluorescence that recovered during washout. Recovery was strongly dependent on Na^+^-Ca^2+^ exchange activity. In cells transfected with NMDA receptor subunits, Cx36-GCaMP revealed transient and concentration-dependent increases in local Ca^2+^ on brief application of glutamate. In HeLa cells, glutamate application increased Cx36-GCaMP tracer coupling through a mechanism that depended in part on Ca^2+^, calmodulin-dependent protein kinase II (CaMKII) activity. This potentiation of coupling did not require exogenous expression of glutamate receptors, but could be accomplished by endogenously expressed glutamate receptors with pharmacological characteristics reminiscent of NMDA and kainate receptors. Analysis of RNA Sequencing data from HeLa cells confirmed expression of NMDA receptor subunits NR1, NR2C, and NR3B. In summary, Cx36-GCaMP is an effective tool to measure changes in the Ca^2+^ microenvironment around Cx36 gap junctions. Furthermore, HeLa cells can serve as a model system to study glutamate receptor-driven potentiation of electrical synapses.

## Visual Abstract

**Figure:**
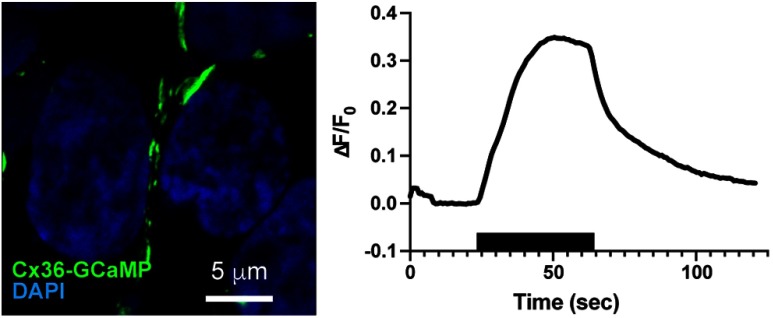

## Significance Statement

We have developed a Connexin 36 (Cx36)-GCaMP3 fusion construct that effectively reports the Ca^2+^ microenvironment in the vicinity of Cx36 gap junctions. This tool will be valuable to investigate the dynamic changes in Ca^2+^ that are responsible for some forms of electrical synapse plasticity. Furthermore, we have discovered that a widely used model system for in vitro studies, the HeLa cell, endogenously expresses glutamate receptors that effectively drive intracellular Ca^2+^, calmodulin-dependent protein kinase II (CaMKII) signaling. This signaling can be exploited in many types of studies.

## Introduction

Electrical synapses provide a means to organize neurons into networks from which high-order activity emerges. Plasticity is a fundamental property of electrical synapses, altering the strength of electrical communication between coupled neurons and potentially playing a large role in regulation of network states (Coulon and Landisman, 2017). Electrical synapse plasticity is also a critical element of microcircuit functions. For example, individual auditory afferent terminals that form mixed chemical and electrical synapses onto Mauthner neurons display a high degree of variability in both electrical and chemical synaptic strength (Smith and Pereda, 2003), influencing the effect of any one auditory neuron on Mauthner cell responses. Both components can be modified by activity, resulting in either potentiation or depression of individual elements, with interdependence of chemical and electrical synapse activity (Pereda et al., 2003b; Smith and Pereda, 2003).

In the fish Mauthner neurons, high-frequency stimulation of the afferent nerve results in potentiation of electrical synapses (Yang et al., 1990; Pereda and Faber, 1996). Activity-dependent potentiation of electrical synapses has also been observed in mammalian AII amacrine cells (Kothmann et al., 2012) and inferior olive neurons (Turecek et al., 2014). Common among these neural networks is the reliance on activation of NMDA receptors, influx of extracellular Ca^2+^ and activation of Ca^2+^, calmodulin-dependent protein kinase II (CaMKII) activity (Pereda et al., 1998, 2003a; Kothmann et al., 2012; Turecek et al., 2014) to potentiate coupling. In retinal AII amacrine cells, the potentiation has been demonstrated to result from CaMKII-dependent phosphorylation of Cx36 (Kothmann et al., 2012). Ca^2+^-dependent depression of electrical synapses has also been reported, depending on NMDA receptors in inferior olive neurons (Mathy et al., 2014) and voltage-dependent Ca^2+^ channels in thalamic reticular neurons (Sevetson et al., 2017), suggesting that the control of coupling by Ca^2+^ entry can be subtle.

Because of the central role of Ca^2+^ signaling in activity-dependent modulation of electrical synapse strength, we wished to investigate the relationship between the Ca^2+^ microenvironment around Cx36 gap junctions and their functional plasticity. To accomplish this, we developed a Cx36 construct fused to the genetically-encoded Ca^2+^ biosensor GCaMP3 (Tian et al., 2009). This construct is an effective tool to examine Ca^2+^ signaling in the context of gap junction functional plasticity.

## Materials and Methods

### Clones

EGFP-N1 vector was obtained from Clontech. A plasmid expressing GCaMP3 in the EGFP-N1 vector was a gift from Loren Looger (Addgene plasmid #22692; Tian et al., 2009). Mouse Connexin 36 (Cx36) cDNA was a gift from Muayyad Al-Ubaidi (University of Oklahoma; Al-Ubaidi et al., 2000). NMDA receptor open reading frame clones were a gift from Vasanthi Jayarman (University of Texas Health Science Center at Houston). These included NR1 in pcDNA 3.1 (NCBI accession #57847), NR2A in pcDNA 3.1 (NCBI accession #286233), NR2B in pcDNA 3.1 (NCBI accession #NM_012574), and NR2C in pRK (NCBI accession #NM_012575.3).

Unless otherwise indicated, restriction enzymes, DNA polymerases and enzymes used for cloning were obtained from New England Biolabs. To produce C-terminal EGFP-tagged Cx36, the mouse Cx36 cDNA was amplified by PCR using Pfu Ultra DNA polymerase (Agilent) with primers T7 and JOB 284 (all primer sequences are listed in Table 1), the latter of which eliminates the stop codon of Cx36 and adds a KpnI site in frame with the EGFP-N1 vector. The PCR product was cloned into EcoRI and KpnI sites of the EGFP-N1 vector by conventional cloning. A C-terminal GCaMP3-tagged Cx36 was produced in a similar fashion using primers T7 and JOB 285, the latter of which eliminates the stop codon of Cx36 and adds a BclI site. The PCR product was digested with SmaI and BclI and cloned into AfeI and BglII sites of the GCaMP3 vector. Resulting clones were fully sequenced. Cx36-GCaMP is available through Addgene (plasmid #123604).

**Table 1:** Primers used for cloning

Name	Sequence	Template
Cx36-EGFP and GCaMP cloning		
T7 extended	GTAATACGACTCACTATAGGGCGAA	MsCx36 cDNA
JOB 284	CCAAACTTGGGTACCCACACATAGGC	MsCx36 cDNA
JOB 285	CCAAACCTTTGATCACACACATAGGC	MsCx36 cDNA
PB513B-1 modification		
KBM 1	AATTAATGACCTGCAGGTCGACGACTGCATAGGGTTAC	pIRES DsRedT3-KR24
KBM 2	GCCGGGATTCTCCTCCACGATCCATTATCATCGTGTTTTTCAA	pIRES DsRedT3-KR24
KBM 3	CGCGCCCGCCGCCCTA	PB513B-1
KBM 4	TGCAGGTCATTAATTAAGGTGGCGTCTAGCGTAGGCG	PB513B-1
Cx36-GCaMP – NR1 dual vector cloning		
KBM 7	AGCGAATTCGAATTTCAGCGATGGGGGAATGG	Cx36-GCaMP
KBM 8	CCGCGGATCCGATTTTACGCCTTAAGATACATTGATGAGTT	Cx36-GCaMP
KBM 405	AGACGCCACCTTAATTAACGGAGCTCATGAGCACCATG	NR1
KBM 406	ATGCAGTCGTCGACCTGCAGGTCCTCAGCTCTCCCTATGACGG	NR1
NR2 cloning		
KBM 11	GCTAGCGAATTCGAATTTCTCTCCACAGGTGTCCACTCC	NR2C
KBM 12	CGGCCGCGGATCCGATTTGGTGCTGCGCCGAATTA	NR2C
KBM 13	GCTAGCGAATTCGAATTTGCTAGCGGCAGATTGGG	NR2A
KBM 14	CGGCCGCGGATCCGATTTCCAGCTGGTTCTTTCCGC	NR2A
KBM 15	GCTAGCGAATTCGAATTTGTGGTGGGCTGAAGACTCCTTA	NR2B
KBM 16	CGGCCGCGGATCCGATTTCCATAGAGCCCACCGCATC	NR2B

A series of clones was created using the PB513B-1 dual expression vector of the Piggybac Transposon System (System Biosciences LLC). The vector was modified to accept a second user-derived sequence by deleting the GFP open reading frame driven by the EF1a promoter by digestion with Bsu36I and BgmBI and inserting a linker containing PacI and SbfI restriction sites followed by an IRES sequence derived from the vector pIRES DsRedT3-KR24 (Moshiri et al., 2008). The modification was completed by three-way assembly of PCR products amplified with Phusion polymerase using primers KBM 1 and KBM 2 for the IRES and primers KBM 3 and KBM 4 to replace a deleted portion of the EF1a promoter. Cold Fusion cloning mix (System Biosciences) was used for the assembly.

The full open reading frame of Cx36-GCaMP, including the SV40 poly A signal, was amplified by PCR using primers KBM 7 and KBM 8 and inserted into the SwaI site following the CMV promoter of the modified PB513 vector using Gibson Assembly. The full coding region of NMDA receptor NR1 was amplified by PCR using primers JOB 405 and JOB 406 and cloned into the PacI and SbfI sites following the EF1a promoter of the same construct to generate a dual-expression construct expressing both NR1 and Cx36-GCaMP. The NR2 subunits were each cloned into the SwaI site of the modified PB513 vector using Gibson Assembly as described above to generate separate single-expression clones. Resulting clones were fully sequenced.

### Cell culture and transfection

Human Embryonic Kidney 293T/17 cells (HEK293, catalog #CRL-11268; ATCC) used for calcium imaging and immunolabeling experiments were grown in DMEM with sodium pyruvate + 10% fetal bovine serum (FBS) with penicillin/streptomycin/fungizone. Cells for all experiments were used between passages 4 and 10 relative to the original cell line obtained from ATCC. Cell culture reagents were obtained from Gibco/ThermoFisher. Cells were plated onto 35-mm culture dishes containing coverslips coated with poly-L-lysine and Laminin (Sigma) and grown to 70–80% confluency. Cells were transiently transfected with Cx36-GCaMP/NR1 and various combinations of NR2 clones in Piggybac vectors, up to a maximum of 4 μg of DNA per dish, using GenePorter H (Genlantis) following the manufacturer’s protocol. For all transfections including clones in the modified PB513 vector, Piggybac transposase (PB200, System Biosciences) was included in the transfection mix. Following transfection, cells were grown in MEM (without essential amino acids) + sodium pyruvate, 10% FBS, penicillin/streptomycin/fungizone and 100 μM APV (DL-2-amino-5-phosphonopentanoic acid; Sigma) to prevent toxicity from activation of transfected NMDA receptors. Cells were cultured for 24 h following transfection, rinsed twice with MEM without APV, and used for experiments.

HeLa cells (catalog #CCL2; ATCC) used for tracer coupling experiments were grown in MEM with essential amino acids + sodium pyruvate, 10% FBS and penicillin/streptomycin/fungizone. Cells for all experiments were used between passages 7 and 20 relative to the original cell line obtained from ATCC. Cells were plated onto tissue culture coated coverslips (ThermoFisher) and grown to 70–80% confluency. Cells were transiently transfected with clones in pcDNA vectors or Piggybac vectors as were HEK293 cells and cultured for 24 h. Before use in experiments, cells were rinsed twice with MEM.

### Immunolabeling

HEK293 cells transfected with Cx36-GCaMP and NMDA receptor clones were fixed in 4% formaldehyde in 0.1 M phosphate buffer (pH 7.5) for 30 min, washed with 0.1 M phosphate buffer, and blocked 1 h in 5% normal donkey serum in 0.1 M phosphate buffer, 0.3% Triton X-100. Cells were then immunolabeled using polyclonal antibodies for rabbit anti-NR2A (PhosphoSolutions; 1:500 dilution), rabbit anti-NR2B (Biosensis; 1:500 dilution), rabbit anti-NR2C (PhosphoSolutions; 1:500 dilution), and rat anti-NMDA NR1 (UC Davis NeuroMab; 1:500 dilution). Cx36-GCaMP labeling was enhanced using anti-GFP (Jackson ImmunoResearch; 1:500 dilution). Cy3 and Cy5-labeled secondary antibodies were purchased from Jackson ImmunoResearch. Imaging was performed on a Zeiss LSM510 Meta confocal microscope using a 40×/1.3 NA objective or a Zeiss LSM780 confocal microscope using a 40×/1.4 NA objective.

### Cell perfusion and imaging

**Figure 1.**
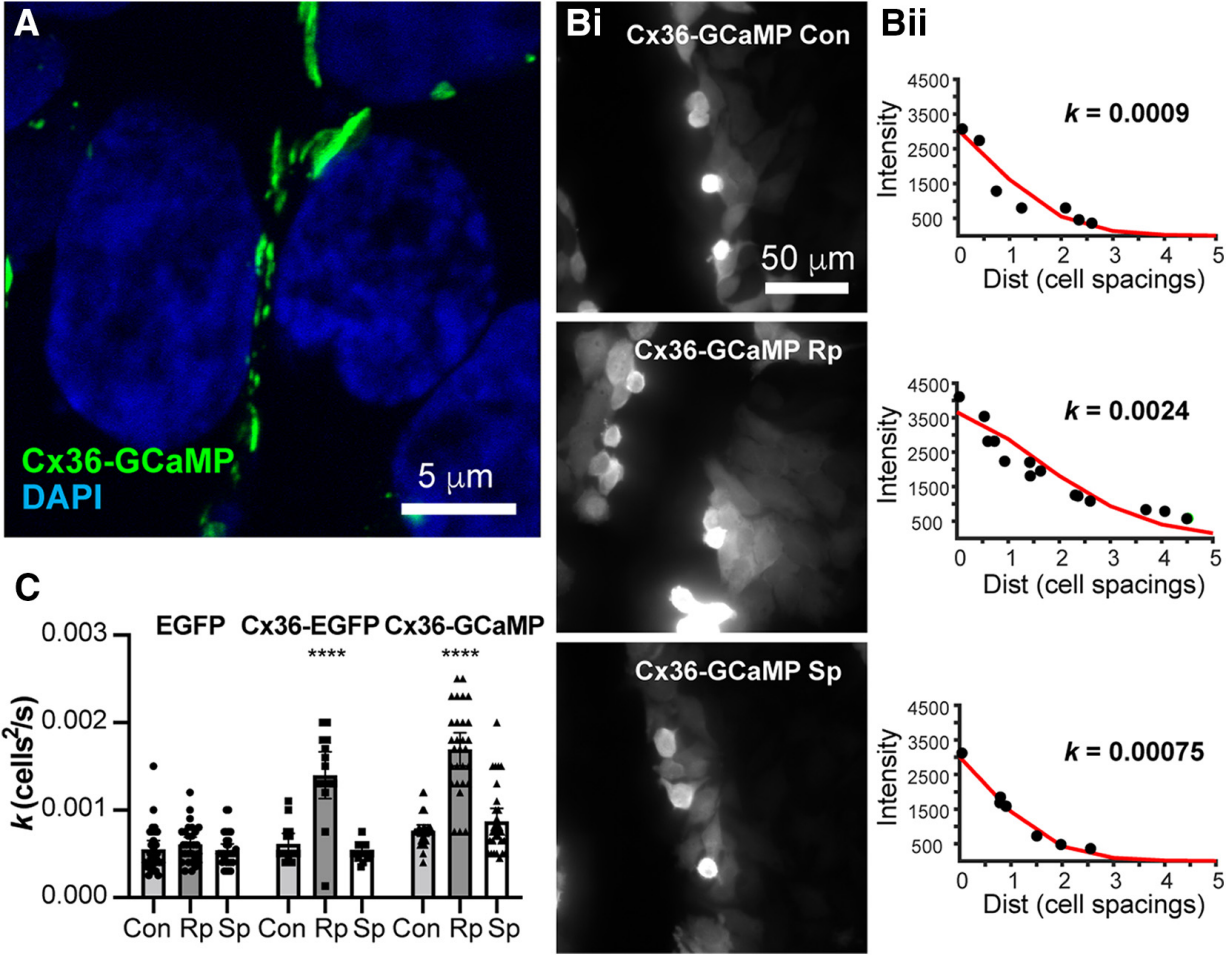
Properties of Cx36-GCaMP. ***A***, Intrinsic fluorescence of Cx36-GCaMP expressed in HEK293 cells. Cx36-GCaMP assembles into gap junctions at cell-cell boundaries. ***B***, Tracer coupling measurements of Cx36-GCaMP expressed in HeLa cells. ***Bi***, Neurobiotin loading and diffusion from the scraped edge in cells in control conditions (Con) or treated with 10 μM PKA inhibitor (Rp) or 10 μM PKA activator (Sp). ***Bii***, Fits of linear compartmental diffusion model to Cy-3 streptavidin fluorescent labeling of Neurobiotin tracer for each of the images shown. Diffusion coefficient *k* determined from the fit, in cells^2^/s, is shown on each graph. ***C***, Mean (bars) diffusion coefficients (*k*) for Neurobiotin tracer coupling in HeLa cells transfected with EGFP, Cx36-EGFP, or Cx36-GCaMP. All data are shown for six (EGFP, Cx36-GCaMP) or three (Cx36-EGFP) experiments. Error bars show 95% confidence limits of the mean; ****p < 0.0001 versus control.

Both HeLa and HEK293 cells were tested in perfusion and imaging experiments. HEK293 cells were superior for this application since most of the transfected Cx36-GCaMP was present at gap junctions and these were well organized at vertical cell-cell interfaces (Fig. 1*A*). HeLa cells displayed a more variable proportion of Cx36-GCaMP at cell-cell versus intracellular membrane compartments, making selection of regions of interest for quantification more difficult. To perform the imaging experiments HEK293 cells on 12-mm coverslips transfected with clones for Cx36-GCaMP, NMDA NR1, and NMDA NR2 subunits (NR2A, NR2B, or NR2C) were placed in perfusion chambers (Model RC-25, Warner Instruments) and perfused with cell maintaining solution (CMS) containing 150 mM NaCl, 6.2 mM KCl, 1. 2 mM NaH_2_PO_4_, 1.2 mM MgSO_4_, 2. 5 mM CaCl_2_, 10 mM glucose, 10 mM HEPES (pH 7.4), and 1 mM glycine (CMS + glycine) at 37°C using a VC^3^ 8-channel gravity perfusion system (ALA Scientific Instruments). Cells were imaged using a Zeiss Axiovert 200 microscope with 40×/0.5 NA objective and an ORCA 100 digital camera (Hamamatsu Photonics) using 470BP40 excitation and 535BP40 emission filters. Live images were captured using HCImage software (Hamamatsu) set to record with 4 × 4 binning and 0.4- to 0.8-s time intervals. Two to five individual gap junctions on each coverslip were marked as regions of interest for recording. Approximately 20-s baseline measurement was obtained under perfusion with CMS + glycine before switching into solutions containing glutamate (30 μM, 100 μM, or 1 mM) for 40 s, followed by return to CMS + glycine. The same perfusion timing was used for applications of 5 μM ionomycin (Fisher Chemical) in CMS + glycine. Ionomycin experiments also included 5 min pre-incubation with 200 nM Na^+^-Ca^2+^ exchanger antagonist SEA 0400 (2-[4-[(2,5-difluorophenyl)methoxy]phenoxy]−5-ethoxybenzenamine; R&D Systems) or 100 nM SR-ER Ca^2+^-ATPase antagonist thapsigargin (R&D Systems) in CMS + glycine, followed by a perfusion experiment with drug in both perfusion solution and ionomycin solution.

Raw fluorescence intensity data from regions of interest were exported from HCImage as tabular data and analyzed in Excel (Microsoft) and Prism (GraphPad). Declining fluorescence intensity of the baseline due to photobleaching was fit with a first-order exponential function or a linear function for each region of interest. This baseline function was used as F_0_ to calculate ΔF/F_0_ without further correction for background fluorescence, since background regions of interest often showed some changes in fluorescence intensity due to scattered signals from nearby fluorescent Cx36-GCaMP structures. For each response, we calculated the peak value of ΔF/F_0_ and the integrated area under the response curve, consisting of the sum of ΔF/F_0_ values for each time point contained within the response normalized to 1-s time intervals.

### Tracer coupling measurements

Gap junction coupling was analyzed by measuring tracer diffusion of Neurobiotin (Vector Laboratories) loaded into cells by scrape loading (el-Fouly et al., 1987; Opsahl and Rivedal, 2000; Risley et al., 2002). HEK293 cells had unacceptably high background tracer coupling due to the presence of endogenous connexins, so tracer coupling experiments were performed in transiently transfected HeLa cells. Cells were incubated in CMS either alone (control) or containing 10 μM protein kinase A antagonist Rp-8-cpt-cAMPs (Axxora LLC), 10 μM protein kinase A agonist Sp-8-cpt-cAMPs (Axxora), 30 μM glutamate + 1 mM glycine, or 100 μM glutamate + 1 mM glycine for 5 min. Additional experiments investigating glutamate receptor contribution to tracer coupling employed the 100 μM glutamate + 1 mM glycine incubation for 5 min with or without 100 nM selective kainate receptor antagonist ACET [(S)−1-(2-amino-2-carboxyethyl)−3-(2-carboxy-5-phenylthiophene-3-yl-methyl)−5-methylpyrimidine-2,4-dione; R&D Systems], 20 μM AMPA/kainate receptor antagonist CNQX (6-cyano-7-nitroquinoxaline-2,3-dione; R&D Systems), 40 μM AMPA and kainate receptor antagonist GYKI 53 655 (1-(4-aminophenyl)−3-methylcarbamyl-4-methyl-3,4-dihydro-7,8-methylenedioxy-5H-2,3-benzodiazepine hydrochloride; R&D Systems), or 20 μM selective NMDA receptor antagonist (R)-CPP [(R)−3-(2-carboxypiperazin-4-yl)-propyl-1-phosphonic acid; R&D Systems]. Fresh solutions of appropriate drug were added to each dish with Neurobiotin at a 0.1% concentration. Cells were scraped with a 26-gauge needle, allowed to incubate 10 min, rinsed three times in CMS, and fixed for 1 h with 4% formaldehyde in 0.1 M phosphate buffer. Fixed cells were rinsed briefly in 0.1 M phosphate buffer, permeabilized with PBS, 0.1% Triton X-100, 0.1% Na azide (PBSTA) for 1 h, and labeled with Cy3-strepavidin (1:500; Jackson ImmunoResearch) for 1.5 h. Coverslips were washed with PBSTA for 1 h and mounted on slides using Vectashield mounting medium (Vector Laboratories) and fluorescently imaged at 40× magnification.

In each experiment, a minimum of five loaded regions along the scraped edge of the cells were identified for complete single cell loading of Neurobiotin with identifiable transfer of tracer away from the loaded cell as an indicator of a coupled region for measurement. Images were collected using HCImage software and analyzed with SimplePCI (Hamamatsu) and MATLAB (MathWorks) software. Intensity of Neurobiotin/Cy3-streptavidin signal was measured in 2-μm circles, with the brightest regions of individual cells selected. Cell-to-cell distance was measured from center to center of adjacent cells. Tracer diffusion was estimated by fitting data from regions of cells extending out from a loaded cell along the scraped edge of the coverslip (Fig. 1*B*) using a linear compartmental diffusion model (Zimmerman and Rose, 1985) as implemented for neural networks (Mills and Massey, 1998; O’Brien et al., 2004). In this model we assume the cells are arranged in a linear compartment chain that is connected by Cx36 gap junctions and may be characterized by a rate-limiting diffusion coefficient *k*. Independent measurements of *k* were made for each of the five to eight loaded regions examined within a single experiment. Three to six experiments were performed and all values of *k* used for comparisons.

### Gene expression analysis

In order to better understand the glutamate receptor and connexin gene expression in HeLa cells, we examined RNASeq dataset GSM759888 (https://www.ncbi.nlm.nih.gov/geo/query/acc.cgi?acc=GSM759888) deposited in GEO (Cabili et al., 2011). The bam file of this dataset was mapped against Hg19 human reference genome using Samtools v1.9 (Genome Research Ltd.; http://www.htslib.org; Li et al., 2009) on macOS v10.13/10.14 to generate a bam indexed (bai) file. The files were analyzed using Integrative Genomics Viewer (IGV) v2.4.14 (Broad Institute and Regents of the University of California; https://software.broadinstitute.org/software/igv/home; Robinson et al., 2011). Each gene of interest was manually searched and an image of read count coverage was exported. IGVtools “count” function was then used to generate the raw read count and normalized read count.

### Statistical analyses

Tracer coupling experiments were performed with control and drug-treated conditions on the same experimental days and batches of cells. Three experiments were performed, except in one case in which a drug treatment did not work, and three additional experiments with control and certain drug treatments were performed. All measurements of *k* were used from the experiments. A criterion of ±3 SDs from the mean was used to exclude outliers among the individual measurements for data analysis. Treatments were compared with one-way or two-way ANOVA with appropriate multiple comparison tests. Tukey’s multiple comparison tests were used when conditions were compared with more than one other condition; Dunnett’s multiple comparisons were used to compare a specific condition in one construct to the same condition in another construct. A summary of all statistical tests performed for this study is presented in Table 2.

**Table 2:** Statistical outcomes

Test/comparison	Multiple comparison/effect size	95% CI of effect size	*p*	n1	exps 1	n2	exps 2
Figure 1							
Two-way ANOVA	Tukey						
EGFP Rp vs Con	5.690 e-005	0.0002499 to –0.0001361	0.7662	29	6	29	6
EGFP Sp vs Con	–1.034 e-005	0.0001827 to –0.0002033	0.9912	29	6	29	6
Cx36-EGFP Rp vs Con	0.0007820	0.001050 to 0.0005136	<0.0001	15	3	15	3
Cx36-EGFP Sp vs Con	–9.667e-005	0.0001717 to –0.0003650	0.6722	15	3	15	3
Cx36-GCaMP Rp vs Con	0.0009282	0.001120 to 0.0007368	<0.0001	29	6	30	6
Cx36-GCaMP Sp vs Con	0.0001067	0.0002964 to –8.309e-005	0.3819	30	6	30	6
Two-way ANOVA	Dunnett						
Cx36-EGFP vs EGFP Con	6.494e-005	0.0002863 to –0.0001564	0.7451	15	3	29	6
Cx36-GCaMP vs EGFP Con	0.0002149	0.0003962 to 3.368e-005	0.0167	30	6	29	6
Cx36-EGFP vs EGFP Rp	0.0007900	0.001011 to 0.0005686	<0.0001	15	3	29	6
Cx36-GCaMP vs EGFP Rp	0.001086	0.001269 to 0.0009034	<0.0001	29	6	29	6
Cx36-EGFP vs EGFP Sp	–2.138e-005	0.0002000 to –0.0002428	0.9680	15	3	29	6
Cx36-GCaMP vs EGFP Sp	0.0003320	0.0005132 to 0.0001507	0.0001	30	6	29	6
Figure 6							
Two-way ANOVA	Tukey						
EGFP 100 Glu vs Con	0.0001200	0.0008372 to –0.0005972	0.9996	15	3	15	3
Cx36-GCaMP 100 Glu vs Con	0.001400	0.002117 to 0.0006828	<0.0001	15	3	15	3
Cx36, NR1 100 Glu vs Con	0.0008867	0.001604 to 0.0001695	0.0052	15	3	15	3
Cx36, NR1, NR2A 100 Glu vs Con	0.001527	0.002244 to 0.0008095	<0.0001	15	3	15	3
100 Glu Cx36-GCaMP vs EGFP	0.001337	0.002054 to 0.0006195	<0.0001	15	3	15	3
100 Glu Cx36, NR1 vs EGFP	0.0007433	0.001461 to 2.617e-005	0.0365	15	3	15	3
100 Glu Cx36, NR1, NR2A vs EGFP	0.001443	0.002161 to 0.0007262	<0.0001	15	3	15	3
100 Glu Cx36-GCaMP vs Cx36, NR1	0.0005933	–0.0001238 to 0.001311	0.1837	15	3	15	3
100 Glu Cx36-GCaMP vs Cx36, NR1, NR2A	–0.0001067	–0.0008238 to 0.0006105	0.9998	15	3	15	3
100 Glu Cx36, NR1 vs Cx36, NR1, NR2A	–0.0007000	–0.001417 to 1.717e-005	0.0610	15	3	15	3
Figure 7							
One-way ANOVA	Tukey						
Glu vs Con	0.001032	0.001242 to 0.0008212	<0.0001	42	6	37	6
Glu+ACET vs Con	–3.643e-006	0.0002442 to –0.0002515	>0.9999	23	3	37	6
Glu+CNQX vs Con	0.0002729	0.0005364 to 9.411e-006	0.0377	19	3	37	6
Glu+GYKI vs Con	0.0002369	0.0005227 to –4.882e-005	0.1651	15	3	37	6
Glu+CPP vs Con	–6.046e-006	0.0002574 to –0.0002695	>0.9999	19	3	37	6
Glu+ACET vs Glu	–0.001035	–0.0007932 to –0.001278	<0.0001	23	3	42	6
Glu+CNQX vs Glu	–0.0007588	–0.0005007 to –0.001017	<0.0001	19	3	42	6
Glu+GYKI vs Glu	–0.0007948	–0.0005139 to –0.001076	<0.0001	15	3	42	6
Glu+CPP vs Glu	–0.001038	–0.0007796 to –0.001296	<0.0001	19	3	42	6
Figure 8							
Two-way ANOVA	Tukey						
EGFP Rp vs Con	–5.000e-005	0.0003220 to –0.0004220	0.9960	18	3	18	3
EGFP Rp+KN93 vs Con	–1.944e-005	0.0003526 to –0.0003914	>0.9999	18	3	18	3
EGFP Glu vs Con	5.408e-005	0.0004315 to –0.0003233	0.9948	17	3	18	3
EGFP Glu+KN93 vs Con	7.173e-005	0.0004492 to –0.0003057	0.9848	17	3	18	3
Cx36-GCaMP Rp vs Con	0.001030	0.001390 to 0.0006693	<0.0001	22	3	17	3
Cx36-GCaMP Rp+KN93 vs Con	0.001068	0.001441 to 0.0006954	<0.0001	19	3	17	3
Cx36-GCaMP Glu vs Con	0.001686	0.002054 to 0.001317	<0.0001	20	3	17	3
Cx36-GCaMP Glu+KN93 vs Con	0.0004001	0.0007605 to 3.976e-005	0.0213	22	3	17	3
Cx36-GCaMP Rp+KN93 vs Rp	3.828e-005	0.0003878 to –0.0003112	0.9982	19	3	22	3
Cx36-GCaMP Glu+KN93 vs Glu	–0.001285	–0.0009407 to –0.001630	<0.0001	22	3	20	3

## Results

### Development of a gap junction calcium sensor

In order to investigate calcium signaling in the microdomain surrounding Cx36 gap junctions we developed a fusion of mouse Cx36 with GCaMP3 at its cytoplasmic C terminus. Similar to fluorescent protein fusions to the C termini of connexins, this construct, called Cx36-GCaMP, formed gap junctions at cell-cell boundaries when transfected into HEK293 cells (Fig. 1*A*) or HeLa cells (data not shown). To determine whether Cx36-GCaMP formed functional gap junctions we performed tracer coupling experiments. Because of high background tracer coupling in HEK293 cells due to endogenous connexins, these experiments were performed in transiently-transfected HeLa cells. HeLa cells transfected with control (EGFP) or connexin constructs (Cx36-EGFP or Cx36-GCaMP) were loaded with Neurobiotin along the edge of a scrape (Fig. 1*Bi*) and tracer diffused to connected cells via gap junctions. Fits of Streptavidin-Cy3 fluorescence versus distance from the loaded cell in cell-to-cell spacings with a linear compartmental diffusion model (Fig. 1*Bii*) allowed calculation of the diffusion coefficient *k* for Neurobiotin tracer transfer. Figure 1*C* shows diffusion coefficients measured in cells transfected with Cx36-GCaMP compared with cells transfected with EGFP-N1 (no gap junction control) and Cx36-EGFP. HeLa cells without an added connexin (EGFP control) support some tracer coupling due to the presence of an endogenous connexin. Tracer coupling in Cx36-GCaMP-transfected HeLa cells was significantly increased by inhibition of endogenous protein kinase A activity with 10 μM Rp-8-cpt-cAMPS [two-way ANOVA with Tukey’s multiple comparison tests; *p* < 0.0001, Con *n* = 30 measurements in six experiments, Rp *n* = 29 measurements in six experiments; mean effect size 0.00093, 95% confidence interval (CI) 0.00074–0.00112), similar to the increase in coupling in Cx36-EGFP-transfected cells (*p* < 0.0001, Con *n* = 15 measurements in three experiments, Rp *n* = 15 measurements in three experiments; mean effect size 0.00078, 95% CI 0.00051–0.00105]. This coupling was significantly greater than in EGFP-transfected cells (two-way ANOVA with Dunnett’s multiple comparison tests: Cx36-GCaMP, *p* < 0.0001, *n* = 29 measurements in six experiments per construct, mean effect size 0.00109, 95% CI 0.00090–0.00127; Cx36-EGFP, *p* < 0.0001, *n* = 15 measurements in three experiments for Cx36-EGFP, *n* = 29 measurements in six experiments for EGFP, mean effect size 0.00079, 95% CI 0.00057–0.00101), indicating that Cx36-GCaMP forms functional gap junction channels similar to those made by Cx36-EGFP. Stimulation of endogenous protein kinase A activity with 10 μM Sp-8-cpt-cAMPS did not significantly change tracer coupling in cells transfected with any of the constructs. None of the treatments significantly affected tracer coupling in EGFP-N1-transfected cells indicating that the endogenous gap junction channels in HeLa cells were not regulated by PKA activity. The pattern of regulation in response to altered PKA activity observed with Cx36-GCaMP is similar to that observed with other Cx36 constructs (Mitropoulou and Bruzzone, 2003; Ouyang et al., 2005), indicating that gap junctions made by this construct are regulated in a normal fashion.

**Figure 2.**
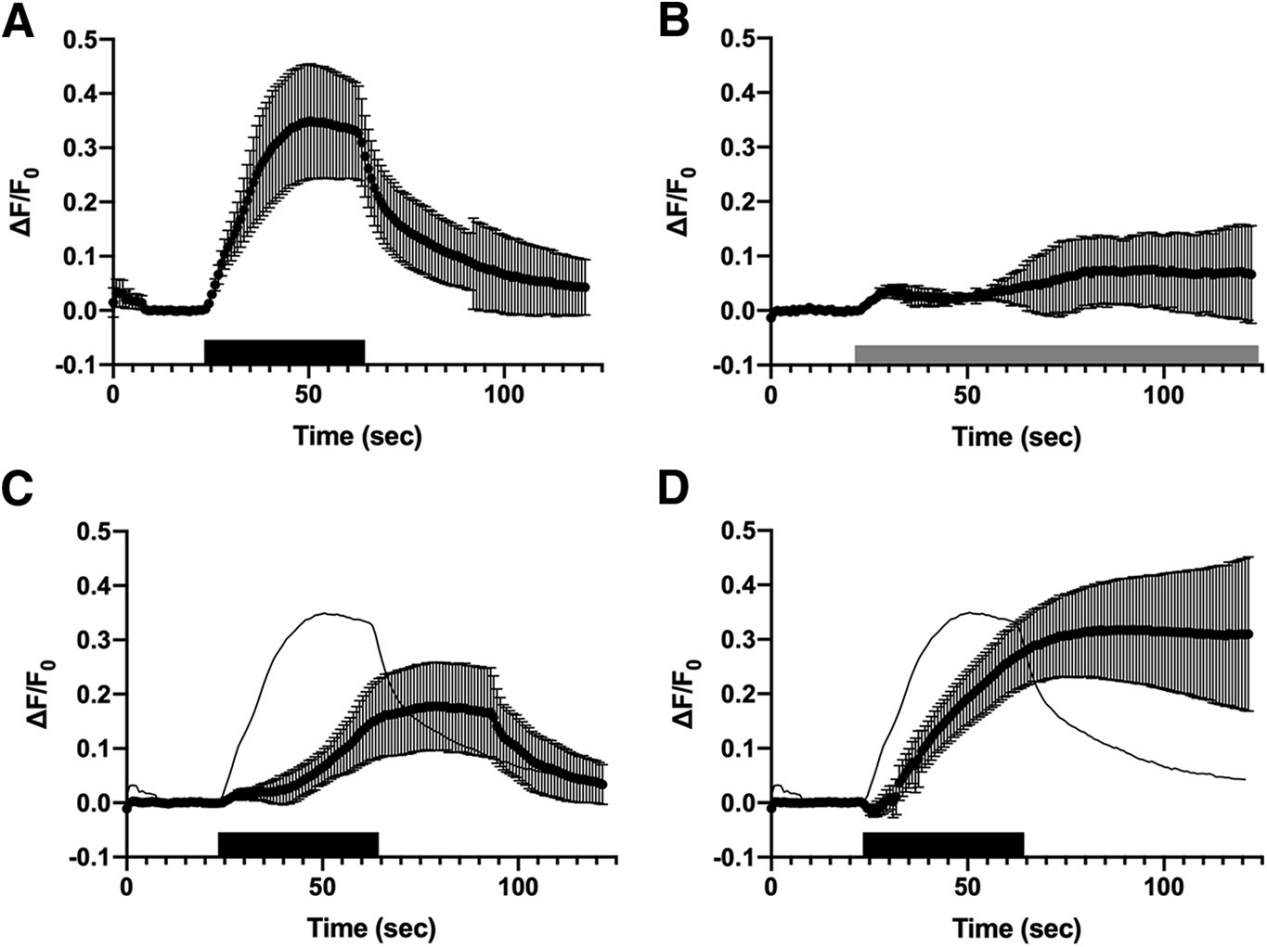
Calcium responses of Cx36-GCaMP. ***A***, Fluorescence response to application of 5 μM ionomycin for 40 s (black bar). Data shown are means of 15 gap junctions in three experiments ± 95% confidence limits of the mean. Note that one of three experiments ended at 92 s, so the final 28 s show 10 gap junctions. ***B***, Fluorescence response to application of 100 nM thapsigargin (gray bar). Data shown are means of five gap junctions in one experiment ± 95% confidence limits of the mean. ***C***, Fluorescence response to application of 5 μM ionomycin (black bar) in the presence of 100 nM thapsigargin. Shown are means of 15 gap junctions in three experiments ± 95% confidence limits of the mean. The mean response to ionomycin in control conditions is shown by the black line for reference. ***D***, Fluorescence response to application of 5 μM ionomycin (black bar) in the presence of 200 nM SEA 0400. Shown are means of 14 gap junctions in three experiments ± 95% confidence limits of the mean. The mean response to ionomycin in control conditions is shown by the black line for reference.

When Cx36-GCaMP-transfected HEK293 cells were superfused with CMS containing 2.5 mM CaCl_2_ and switched to CMS plus 5 μM ionomycin, Cx36-GCaMP gap junctions displayed robust increases in fluorescence (Fig. 2*A*; ionomycin application indicated by the black bar) indicating a rise in intracellular calcium in the vicinity of the gap junctions (*n* = 15 gap junctions in three experiments). Ca^2+^ remained elevated while ionomycin perfusion continued (Fig. 2*A*), but recovered rapidly on washout in normal CMS. To better understand the etiology of the recovery we investigated the influence of the SR/ER calcium ATPase and the Na^+^/Ca^2+^ exchanger on calcium dynamics. Perfusion of the SR/ER calcium ATPase inhibitor thapsigargin (100 nM; Fig. 2*B*, gray bar) caused an immediate, small increase in intracellular Ca^2+^, followed by a more variable delayed rise in intracellular Ca^2+^ (*n* = 5 gap junctions in one experiment)_._ Application of 5 μM ionomycin after 5-min pretreatment with thapsigargin (Fig. 2*C*; *n* = 15 gap junctions in three experiments) resulted in a delayed and reduced relative rise in intracellular Ca^2+^ with delayed recovery (compare response to the mean ionomycin response in control conditions shown by the black line; ionomycin treatment indicated by the black bar). Note that the intracellular Ca^2+^ used as the baseline F_0_ is elevated, so the peak Ca^2+^ may not be substantially lower than in control conditions. Treatment with the Na^+^/Ca^2+^ exchange inhibitor SEA 0400 (200 nM) also delayed the rise in intracellular Ca^2+^ and prevented recovery (Fig. 2*D*; *n* = 14 gap junctions in three experiments). Thus, both Ca^2+^ stores and plasma membrane Ca^2+^ handling significantly influence the kinetics of Ca^2+^ rise and recovery.

### NMDA receptor activation stimulates Ca^2±^ signals at Cx36-GCaMP gap junctions

**Figure 3.**
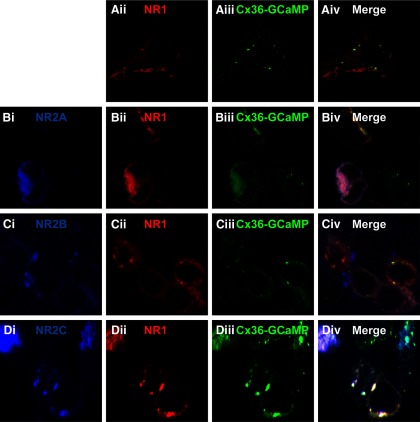
Immunofluorescence labeling of Cx36-GCaMP and NMDA receptors transfected into HEK293 cells. For each transfection combination, Cx36-GCaMP is shown in green, NR1 in red, and NR2x in blue. ***A***, Cx36-GCaMP + NR1. ***B***, Cx36-GCaMP + NR1 + NR2A. ***C***, Cx36-GCaMP + NR1 + NR2B. ***D***, Cx36-GCaMP + NR1 + NR2C. Well-formed gap junctions at cell-cell boundaries were used for live imaging experiments, while overexpressing cells with diffusely distributed Cx36-GCaMP were avoided.

To investigate the coupling of NMDA receptor activation to calcium signals in the vicinity of Cx36 we transfected HEK293 cells with Cx36-GCaMP, NMDA receptor NR1 subunit and one of the NMDA receptor NR2 subunits NR2A, NR2B or NR2C. Figure 3 shows immunostaining of HEK293 cells transfected with Cx36-GCaMP and NR1 (Fig. 3*A*), or these two plus NR2A (Fig. 3*B*), NR2B (Fig. 3*C*), or NR2C (Fig. 3*D*). Expression level did vary among cells, and expression of very high levels of protein in a cell frequently caused delocalization of Cx36-GCaMP away from gap junctions. Only well-formed gap junctions were used for imaging Ca^2+^ responses, despite quite robust responses observed in cells diffusely expressing Cx36-GCaMP.

**Figure 4.**
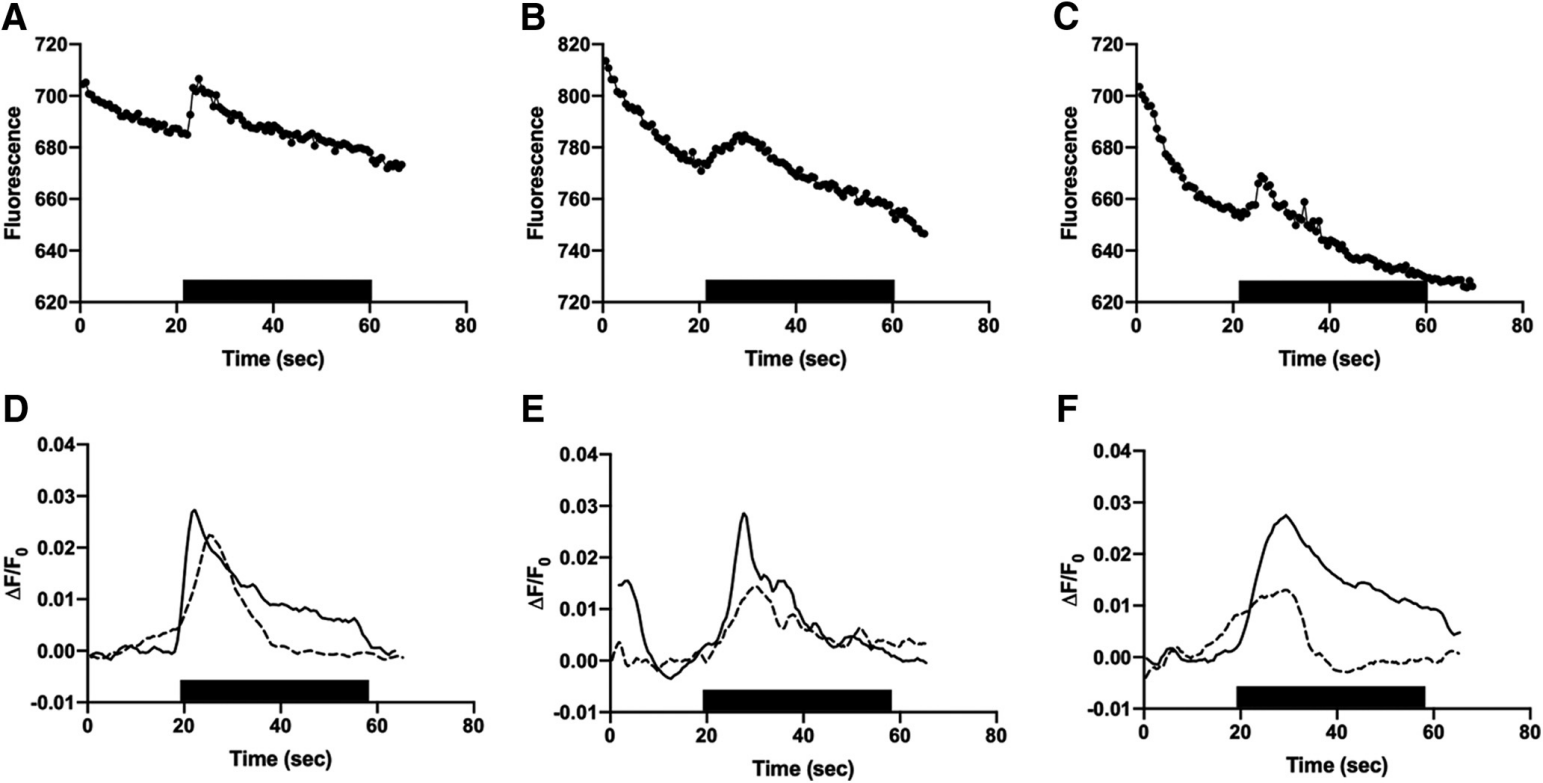
Cx36-GCaMP gap junction responses to glutamate application. Shown in ***A–C*** are representative single gap junction raw fluorescence responses to bath application of 100 μM glutamate (black bar) in HEK293 cells expressing NMDA receptors containing NR1 and NR2A (***A***), NR2B (***B***), or NR2C (***C***). Baseline subtracted average responses to 30 μM (dashed lines) and 100 μM (solid lines) glutamate are shown below in ***D–F***. ***D***, 30 μM NR2A, average of eight gap junctions from two experiments; 100 μM NR2A, average of five gap junctions from one experiment. ***E***, 30 μM NR2B, average of seven gap junctions from two experiments; 100 μM NR2B, average of four gap junctions from one experiment. ***F***, 30 μM NR2C, average of six gap junctions from two experiments; 100 μM NR2C, average of 11 gap junctions from three experiments.

**Figure 5.**
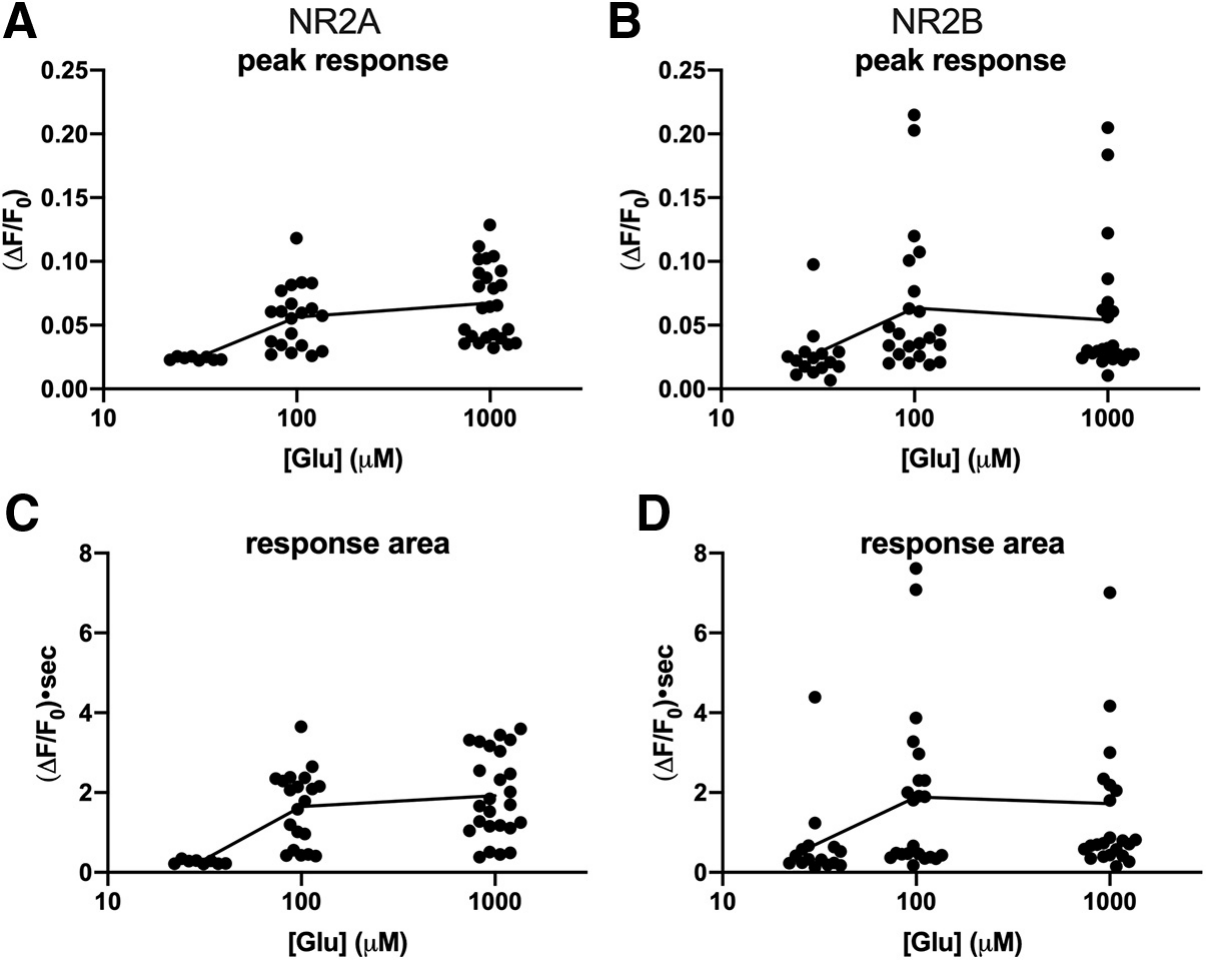
Glutamate concentration-response relationships of Cx36-GCaMP gap junctions in HEK293 cells expressing NR2A-containing and NR2B-containing NMDA receptors. ***A***, ***B***, Baseline-subtracted fluorescence peak response for NR2A (***A***) and NR2B-containing (***B***) cells. ***C***, ***D***, Integrated area under the response curve for NR2A (***C***) and NR2B-containing (***D***) cells. All data are shown for 8–25 gap junctions from two to eight experiments per condition. The black lines connect the mean responses.

Triple-transfected cells were superfused with CMS and switched for 40 s into a solution containing 30 μM, 100 μM, or 1 mM glutamate. CMS in all conditions contained 1 mM glycine to act as a co-agonist for NMDA receptor activation. Figure 4*A–C* shows example raw single Cx36-GCaMP gap junction responses to 100 μM glutamate stimulation. NMDA receptors containing NR2A, NR2B or NR2C all drove transient increases in GCaMP fluorescence, indicative of Ca^2+^ increases in the microenvironment surrounding the gap junction. Baseline-subtracted average responses from 4 to 11 gap junctions are shown in Figure 4*D–F*. All three types of NMDA receptors drove robust transient increases in local Ca^2+^ within the first few seconds of glutamate application followed by gradual declines. There were no consistent differences in the kinetics of Ca^2+^ signals recorded at Cx36-GCaMP gap junctions in response to glutamate stimulation of NMDA receptors containing NR2A, NR2B or NR2C. Figure 5*A*,*B* shows concentration-response relationships of the response peak of 8–25 gap junctions in two to eight experiments in cells expressing NR1 and NR2A or NR2B to 30 μM, 100 μM, and 1 mM glutamate. Both NMDA receptor types drove concentration-dependent increases in peak response that were largely similar to each other, with both appearing to saturate between 100 μM and 1 mM glutamate. Because changes in signaling to the gap junction are likely to depend on the total Ca^2+^ encountered during NMDA receptor responses, we also compared integrated areas under the response curve (Fig. 5*C*,*D*) during the course of the responses. These also showed concentration-dependent increases that saturated between 100 μM and 1 mM glutamate.

### Glutamate receptor activation increases coupling

**Figure 6.**
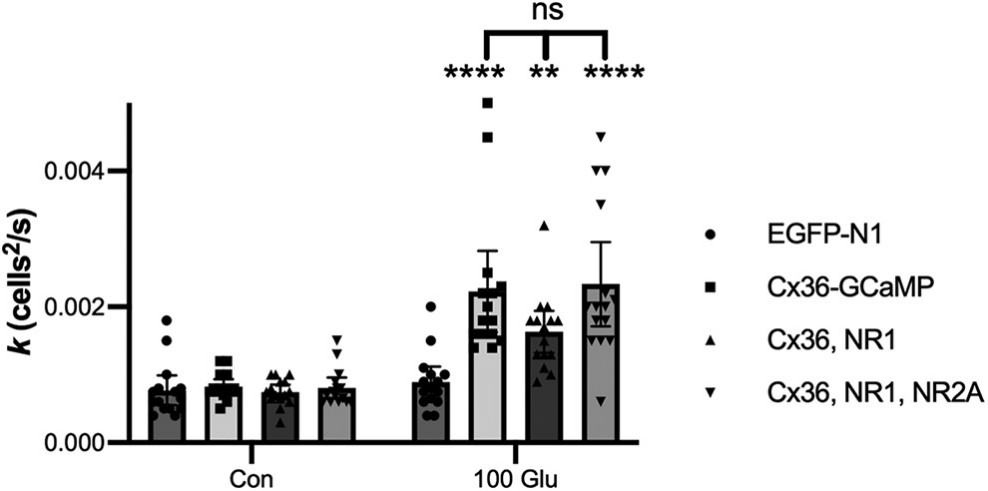
Effects of glutamate application on tracer coupling in HeLa cells expressing EGFP or Cx36-GCaMP with or without added NMDA receptor subunits. The diffusion coefficient (*k*) for Neurobiotin tracer diffusion is shown for 5-min preincubation plus 10-min tracer diffusion time in control media (Con) or control media plus 100 μM glutamate (100 Glu). All data are shown from three experiments; bars show mean values; error bars show 95% confidence limits of the mean; **p < 0.01, ****p < 0.0001 versus same transfection composition in control media.

NMDA receptor activation in retinal AII amacrine cells (Kothmann et al., 2012) and inferior olive neurons (Turecek et al., 2014) increases Cx36 coupling through Ca^2+^ and CaMKII-dependent phosphorylation of Cx36. To examine whether NMDA receptor activation can control coupling in Cx36-GCaMP, we examined the effect of 5-min incubation of 100 μM glutamate on tracer coupling in HeLa cells transiently transfected with Cx36-GCaMP, Cx36-GCaMP plus NMDA receptor subunit NR1, or Cx36-GCaMP plus NMDA receptor subunits NR1 and NR2A. EGFP transfection served as a no gap junction control. Figure 6 shows that 100 μM glutamate significantly increased coupling in cells expressing intact NMDA receptors consisting of NR1 and NR2A (two-way ANOVA with Tukey’s multiple comparison tests; *p* < 0.0001, mean effect size 0.00153, 95% CI 0.00081–0.00224; *n* = 15 measurements per condition in three experiments). There was no change in coupling in EGFP-transfected controls (two-way ANOVA; *p* = 0.999; *n* = 15). Surprisingly, 100 μM glutamate significantly increased coupling when only the NR1 subunit, which does not by itself form a functional NMDA receptor, was transfected with Cx36-GCaMP (two-way ANOVA; *p* = 0.0052; mean effect size 0.00089, 95% CI 0.00017–0.00160; *n* = 15). Very much to our surprise, HeLa cells transfected only with Cx36-GCaMP also showed a significant increase in coupling with the 100 μM glutamate incubation (two-way ANOVA; *p* < 0.0001, mean effect size 0.00140, 95% CI 0.00068–0.00212; *n* = 15). Furthermore, there were no significant differences in the 100 μM glutamate condition between cells transfected with Cx36-GCaMP + NR1 and cells transfected with Cx36-GCaMP alone or with Cx36-GCaMP + NR1 + NR2A (two-way ANOVA), and all were significantly greater than the EGFP control (Cx36-GCaMP *p* < 0.0001, *n* = 15, mean effect size 0.00134, 95% CI 0.00062–0.00205; Cx36-GCaMP + NR1 *p* = 0.037, *n* = 15, mean effect size 0.00074, 95% CI 0.00003–0.00146; Cx36-GCaMP + NR1 + NR2A *p* < 0.0001, *n* = 15, mean effect size 0.00144, 95% CI 0.00073–0.00216). These results suggest that HeLa cells express an endogenous glutamate receptor that contributes to signaling that increases coupling in Cx36-GCaMP.

**Figure 7.**
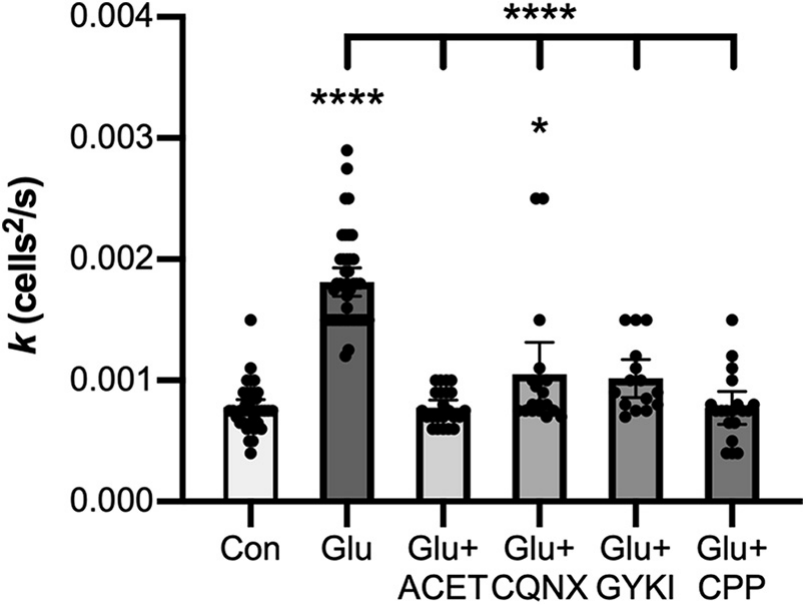
Pharmacological characteristics of endogenous glutamate receptors in HeLa cells expressing Cx36-GCaMP. The diffusion coefficient (*k*) for Neurobiotin tracer diffusion is shown for 5-min preincubation plus 10-min tracer diffusion time in control media (Con), control media plus 100 μM glutamate (Glu), or control media plus 100 μM glutamate plus 100 nM ACET (Glu+ACET), 10 μM CNQX (Glu+CNQX), 40 μM GYKI 53 655 (Glu+GYKI), or 10 μM CPP (Glu+CPP). All data are shown from six (Con, Glu) or three experiments; bars show mean values; error bars show 95% confidence limits of the mean; *p < 0.05, ****p < 0.0001 versus control condition; comparison of each drug versus 100 μM Glu, shown by the bracket, yielded *p* < 0.0001 for all.

We further characterized the glutamate receptors endogenously expressed in HeLa cells through pharmacological experiments using the effect of 5-min incubation with 100 μM glutamate on tracer coupling of Cx36-GCaMP-transfected cells as an assay. Figure 7 shows that several selective and poorly selective glutamate receptor inhibitors prevented the increase in coupling caused by incubation with 100 μM glutamate (one-way ANOVA with Tukey’s multiple comparison tests). Each of the following significantly reduced coupling below the 100 μM glutamate condition: 100 nm of the selective kainate receptor antagonist ACET (*p* < 0.0001, Glu *n* = 42 measurements in six experiments, Glu+ACET *n* = 23 measurements in three experiments, mean effect size −0.00104, 95% CI −0.00079 to −0.00128), 10 μM of the poorly selective AMPA/kainate receptor antagonist CNQX (*p* < 0.0001, Glu *n* = 42 measurements in six experiments, Glu+CNQX *n* = 19 measurements in three experiments, mean effect size −0.00076, 95% CI −0.00050 to −0.00102), 40 μM of the poorly selective AMPA/kainate receptor antagonist GYKI 53 655 (*p* < 0.0001, Glu *n* = 42 measurements in six experiments, Glu+GYKI *n* = 15 measurements in three experiments, mean effect size −0.00080, 95% CI −0.00051 to −0.00108), or 10 μM of the selective NMDA receptor antagonist CPP (*p* < 0.0001, Glu *n* = 42 measurements in six experiments, Glu+CPP *n* = 19 measurements in three experiments, mean effect size −0.00104, 95% CI −0.00078 to −0.00130). Of these treatments, only CNQX did not fully block the increase caused by 100 μM glutamate, yielding a very slight increase in coupling (*p* = 0.038, Con *n* = 39 measurements in six experiments, Glu+CNQX *n* = 19 measurements in three experiments, mean effect size 0.00027, 95% CI 0.00001–0.00054). This indicates that glutamate receptors in HeLa cells that influence Cx36 coupling have pharmacological characteristics of kainate and NMDA receptors, and may as well have some characteristics of AMPA receptors.

To further investigate the glutamate receptor expression in HeLa cells, we analyzed HeLa RNA Sequence dataset GSM759888 deposited in GEO (Cabili et al., 2011), searching for all of the annotated human ionotropic and metabotropic glutamate receptor genes. Sequence reads were found aligning to a kainate receptor Grik1a antisense transcript, and to transcripts of NMDA receptor subunit genes Grin1 (NR1), Grin2c (NR2C), and Grin3b (NR3B; Table 3). No other glutamate receptor gene transcripts were detected. This further confirms that NMDA receptor genes are expressed in HeLa cells, but does not support the expression of kainate-type receptors, as suggested by the pharmacological properties of the glutamate-driven coupling changes.

**Table 3:** HeLa gene expression analysis

Glutamate receptor genes			
Gene		Raw count	Normalized read count
Grik1-As2	GRIK1 antisense RNA 2	1	0.033173785
Grin1	Glutamate ionotropic receptor NMDA type subunit 1	1.531111111	0.050792751
Grin2c	Glutamate ionotropic receptor NMDA type subunit 2C	1	0.033173785
Grin3b	Glutamate ionotropic receptor NMDA type subunit 3B	1.363636364	0.04523698
Grm1	Glutamate metabotropic receptor 1	0	0
Grm2	Glutamate metabotropic receptor 2	0	0
Grm3	Glutamate metabotropic receptor 3	0	0
Grm4	Glutamate metabotropic receptor 4	0	0
Grm5	Glutamate metabotropic receptor 5	0	0
Grm5-As1	GRM5 antisense RNA 1	0	0
Grm6	Glutamate metabotropic receptor 6	0	0
Grm7	glutamate metabotropic receptor 7	0	0
Grm7-As1	GRM7 antisense RNA 1	0	0
Grm7-As2	GRM7 antisense RNA 2	0	0
Grm7-As3	GRM7 antisense RNA 3	0	0
Grm8	Glutamate metabotropic receptor 8	0	0
Grik1	Glutamate ionotropic receptor kainate type subunit 1	0	0
Grik1-As1	GRIK1 antisense RNA 1	0	0
Grik2	Glutamate ionotropic receptor kainate type subunit 2	0	0
Grik3	Glutamate ionotropic receptor kainate type subunit 3	0	0
Grik4	Glutamate ionotropic receptor kainate type subunit 4	0	0
Grik5	Glutamate ionotropic receptor kainate type subunit 5	0	0
Gria1	Glutamate ionotropic receptor AMPA type subunit 1	0	0
Gria2	Glutamate ionotropic receptor AMPA type subunit 2	0	0
Gria3	Glutamate ionotropic receptor AMPA type subunit 3	0	0
Gria4	Glutamate ionotropic receptor AMPA type subunit 4	0	0
Grid1	Glutamate ionotropic receptor delta type subunit 1	0	0
Grid1-As1	GRID1 antisense RNA 1	0	0
Grid2	Glutamate ionotropic receptor delta type subunit 2	0	0
Grin2a	Glutamate ionotropic receptor NMDA type subunit 2A	0	0
Grin2b	Glutamate ionotropic receptor NMDA type subunit 2B	0	0
Grin2d	Glutamate ionotropic receptor NMDA type subunit 2D	0	0
Grin3a	Glutamate ionotropic receptor NMDA type subunit 3A	0	0
Connexin genes			
Gene		Raw count	Normalized read count
GJA1	Gap junction protein alpha 1 (Cx43)	14.45	0.479361193
GJA9-MYCBP	GJA9 (Cx59)-MYCBP readthrough transcript	16.58024691	0.550029546
GJB3	Gap junction protein beta 3 (Cx31)	1.123076923	0.037256712
GJC1	Gap junction protein gamma 1 (Cx45)	5.186435986	0.172053712
GJC2	Gap junction protein gamma 2 (Cx47)	2.0465	0.067890151
GJD3	Gap junction protein delta 3 (Cx31.9)	2.713594595	0.090020204
GJA3	Gap junction protein alpha 3 (Cx46)	0	0
GJA4	Gap junction protein alpha 4 (Cx37)	0	0
GJA5	Gap junction protein alpha 5 (Cx40)	0	0
GJA8	Gap junction protein alpha 8 (Cx50)	0	0
GJA10	Gap junction protein alpha 10 (Cx62)	0	0
GJB1	Gap junction protein beta 1 (Cx32)	0	0
GJB2	Gap junction protein beta 2 (Cx26)	0	0
GJB4	Gap junction protein beta 4 (Cx30.3)	0	0
GJB5	Gap junction protein beta 5 (Cx31.1)	0	0
GJB6	Gap junction protein beta 6 (Cx30)	0	0
GJB7	Gap junction protein beta 7 (Cx25)	0	0
GJC3	Gap junction protein gamma 3 (Cx29)	0	0
GJD2	Gap junction protein delta 2 (Cx36)	0	0
GJD4	Gap junction protein delta 4 (Cx40.1)	0	0

We also examined the expression of connexin genes to gain insight into the connexin background that contributes to coupling in HeLa cells (Table 3). Substantial numbers of reads mapped to Gja1 (Cx43) and Gjc1 (Cx45) genes, and a smaller number of reads mapped to Gja9 (Cx59)-MYCB readthrough transcript, Gjb3 (Cx31), Gjc2 (Cx47), and Gjd3 (Cx31.9). Thus, there is likely a mixed background of connexins contributing to the coupling detected in control (EGFP-transfected) cells, and as well in those transfected with connexin constructs.

**Figure 8.**
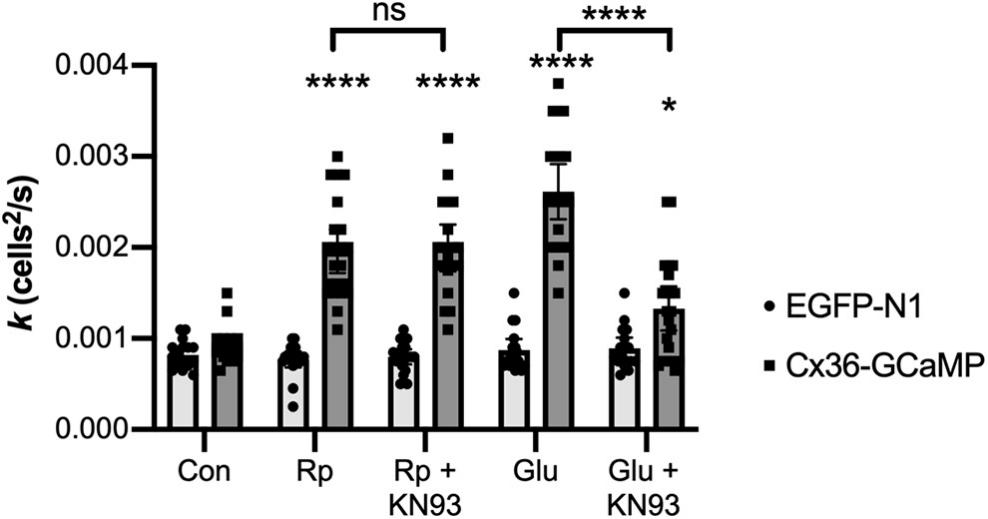
Protein kinase signaling pathways responsible for potentiation of Cx36-GCaMP tracer coupling by 100 μM glutamate in HeLa cells. The diffusion coefficient (*k*) for Neurobiotin tracer diffusion is shown using the same experimental paradigm as in Fig. 6. Rp = PKA inhibitor (10 μM); KN-93 = CaMKII inhibitor (10 μM); Glu = 100 μM glutamate. All data are shown from three experiments; bars show mean values; error bars show 95% confidence limits of the mean; *p < 0.05, ****p < 0.0001; ns = not significant. Symbols above bars represent comparison versus same transfected construct in control media; symbols above brackets represent comparison of the conditions underlying the ends of the brackets.

Because glutamate receptor signaling that increases coupling in Cx36 gap junctions depends, at least in part, on CaMKII activity (Pereda et al., 1998; Kothmann et al., 2012), we examined whether CaMKII was involved in the glutamate-driven increase in Cx36-GCaMP coupling in HeLa cells. Because of the presence of endogenous glutamate receptors that influenced coupling, we did not add additional NMDA receptor subunits. Figure 8 shows tracer coupling of HeLa cells transfected with Cx36-GCaMP or with EGFP. Treatment with 10 μM CaMKII inhibitor KN-93 significantly reduced the increase in coupling caused by 100 μM glutamate incubation (two-way ANOVA with Tukey’s multiple comparison tests: *p* < 0.0001, Glu *n* = 20 measurements in three experiments, Glu+KN-93 *n* = 22 measurements in three experiments, mean effect size −0.00129, 95% CI −0.00094 to −0.00163), although coupling was still slightly enhanced compared with control (*p* = 0.021, Con *n* = 17 measurements in three experiments, Glu+KN-93 *n* = 22 measurements in three experiments, mean effect size 0.00040, 95% CI 0.00041–0.00076). In contrast, while coupling was significantly increased by inhibition of PKA with 10 μM Rp-8-cpt-cAMPS (*p* < 0.0001, Con *n* = 17 measurements in three experiments, Rp *n* = 22 measurements in three experiments, mean effect size 0.00103, 95% CI 0.00067–0.00139), 10 μM KN-93 did not block this increase (*p* = 0.998, Rp *n* = 22 measurements in three experiments, Rp+KN-93 *n* = 19 measurements in three experiments). Thus, the enhancement of coupling caused by activation of endogenous glutamate receptors depended in part on CaMKII, while the coupling revealed by PKA inhibition did not.

## Discussion

Plasticity is a fundamental property of electrical synapses (Curti and O'Brien, 2016) and a great deal of this plasticity results from activity-dependent processes (Pereda et al., 2013; Haas et al., 2016). Calcium signaling has been identified as a critical element of electrical synapse activity-dependent plasticity, controlling protein kinase and phosphatase activities that modify synapse strength (Haas et al., 2016; Coulon and Landisman, 2017; Sevetson et al., 2017; O’Brien, 2019). We have designed a gap junction-localized calcium sensor to study the microenvironment that is most relevant for regulation of electrical synapse plasticity in hopes to further the understanding of dynamic regulation of electrical synapse strength.

Cx36-GCaMP responds robustly to influx of extracellular Ca^2+^ and reports dynamic changes in its concentration in the immediate vicinity of the gap junction. The dynamic nature of the Ca^2+^ microenvironment is particularly apparent in the response to administration of ionomycin to HEK cells expressing Cx36-GCaMP (Fig. 2). While Ca^2+^ rises to a peak shortly after onset of ionomycin administration, its level plateaus and is rapidly reduced on washout by cellular processes, which include both extrusion via Na^+^-Ca^2+^ exchangers and sequestration in intracellular compartments such as endoplasmic reticulum and mitochondria. It is likely that the plasma membrane Ca^2+^-ATPase, which we did not examine, also plays a significant role in extrusion of Ca^2+^ from the cell. Intracellular stores in the ER play a complex role. Inhibiting the SERCA pump with thapsigargin raised intracellular Ca^2+^ only a small amount, but potently damped the rate of rise of Ca^2+^ on ionomycin addition. This suggests that the rise in control conditions includes elements of Ca^2+^-induced Ca^2+^ release from the ER stores, or that the elevated cytoplasmic Ca^2+^ has activated the plasma membrane Ca^2+^-ATPase or Na^+^-Ca^2+^ exchanger. It is likely that all of these processes are involved in the dynamics of Ca^2+^ in the vicinity of the Cx36 gap junctions.

Pertinent to understanding activity-dependent plasticity are the responses of Cx36-GCaMP to glutamate administration in the presence of NMDA receptors. All three types of NMDA receptors tested, those containing NR2A, NR2B, and NR2C, drove qualitatively similar Ca^2+^ responses at Cx36-GCaMP gap junctions. Responses peaked in the first few seconds of glutamate administration and often declined in the continued presence of glutamate. The decline no doubt reflects both the desensitization kinetics of the NMDA receptors and the Ca^2+^ removal processes described above. Despite these processes, Ca^2+^ remained elevated above the baseline level throughout the duration of the glutamate application for glutamate concentrations of 100 μM or higher. This suggests that signaling driven by elevated Ca^2+^ may be activated continuously when glutamate is present, at least at higher concentrations.

Kothmann et al. (2012) demonstrated that activation of non-synaptic NMDA receptors associated with Cx36 drove an increase in CaMKII activity, resulting in phosphorylation of Cx36 and increased coupling. In an analogous fashion, activation of synaptic NMDA receptors drives potentiation of the electrical synapses in the Mauthner cell mixed synapses through CaMKII activity (Pereda et al., 1998). Phosphorylation of the connexins involved has not been evaluated in that system, but the presence of Cx35 (the fish orthologue of mammalian Cx36) and the highly homologous Cx34.7 at these synapses (Pereda et al., 2003a; Rash et al., 2013) strongly suggests that connexin phosphorylation is the mechanism responsible for activity-dependent potentiation. Such activity-dependent potentiation could be mimicked in the HeLa cell expression system. Tracer coupling supported by Cx36-GCaMP was enhanced by glutamate application in HeLa cells expressing NR2A-containing NMDA receptors. In a surprising twist, tracer coupling was also enhanced by glutamate application in HeLa cells in which no NMDA receptor subunits had been transfected. The increase in coupling depended on CaMKII activity, as would be predicted by a Ca^2+^-mediated response, suggesting the presence of an endogenous glutamate receptor that supports Ca^2+^ influx in HeLa cells. Furthermore, the CaMKII activity in HeLa cells was not constitutive. The intervention routinely performed in our group of inhibiting PKA activity with Rp-8-cpt-cAMPS, which appears to increase coupling by reducing PKA-stimulated protein phosphatase 2A activity (Kothmann et al., 2009; Yoshikawa et al., 2017), increased coupling in a manner independent of CaMKII activity. Thus, coupling could be increased by constitutive activity of an as yet unidentified protein kinase as well as through glutamate-inducible CaMKII activity. The latter pathway provides a useful model system to investigate activity-dependent potentiation of coupling.

In Neuro2A cells, Del Corsso et al. (2012) found CaMKII activation to be responsible for a gradual increase in Cx36-mediated coupling as a result of breaking into a cell pair with patch pipettes. The stimulus for this “run-up” is unclear, although a transient increase in intracellular Ca^2+^ on break-in was proposed. Thus, in both expression systems (Del Corsso et al., 2012 and this study) and natural systems (Pereda et al., 1998; Kothmann et al., 2012; Turecek et al., 2014), CaMKII activity supports potentiation of coupling. CaMKII is a potent and central regulator of synaptic activity, affecting both AMPA receptor conductance and addition of AMPA receptors to post-synaptic densities (Coultrap and Bayer, 2012). Its role at electrical synapses seems to be comparable. CaMKII phosphorylates Cx36 directly and indeed binds to Cx36 in a manner similar to its binding to NR2B (Alev et al., 2008). In addition, CaMKII is proposed to support the maintenance or insertion of Cx36 channels, since its inhibition reduced the number of gap junctions on inferior olive neurons (Bazzigaluppi et al., 2017). Thus, CaMKII has attributes that make it likely to be a component of the complex associated with electrical synapses.

CaMKII is comprised of four isoforms, each with several splice variants, that have distinct cell type distributions, activity and protein binding characteristics, and effects on various aspects of learning and memory (Zalcman et al., 2018). The distributions of the four major isoforms in the context of Cx36 has been examined in the retina (Tetenborg et al., 2017). Interestingly, the major neuronal isoform CaMKIIα showed virtually no association with Cx36, while the neuronal isoform CaMKIIβ and the widespread isoform CaMKIIδ were associated with Cx36 gap junctions in a somewhat cell-type selective manner (Tetenborg et al., 2017, 2019). In AII amacrine cells, in which the CaMKII-driven potentiation of Cx36 coupling depends on Cx36 phosphorylation (Kothmann et al., 2012), CaMKIIδ was the major isoform present (Tetenborg et al., 2017). This isoform supports the highest rate of autophosphorylation of the four (Gaertner et al., 2004), which leads to autonomous activity, albeit considerably lower in the absence of Ca^2+^-calmodulin binding (Coultrap and Bayer, 2012). In this manner, transient glutamate receptor signaling at Cx36 gap junctions can activate CaMKII to produce sustained phosphorylating activity that potentiates the electrical synapse. In contrast, Bazzigaluppi et al. (2017) have found that knockout of CaMKIIβ, but not CaMKIIα, reduced the total number of Cx36 gap junctions in inferior olive neurons, suggesting that the CaMKIIβ isoform is important for maintenance or insertion of Cx36 in gap junctions. Thus, the means through which CaMKII enhances coupling may differ according to the isoform involved.

Potentiation is not the only outcome of neuronal activity surrounding electrical synapses. In contrast to the NMDA receptor and CaMKII-dependent potentiation of coupling in inferior olive neurons resulting from NMDA application or selective paired-pulse presynaptic stimulation (Turecek et al., 2014), low frequency presynaptic stimulation resulted in lasting depression (Mathy et al., 2014). This depression depended on NMDA receptors, intracellular Ca^2+^ and CaMKII activity, although it is not clear how the electrical synaptic depression would be achieved. More in keeping with current understanding is the depression of thalamic reticular neuron electrical synapses by burst activity (Sevetson et al., 2017). In these neurons, Ca^2+^ entry through T-type voltage-gated Ca^2+^ channels activates calcineurin, with the predicted reduction in Cx36-mediated coupling. The bidirectional modulation of electrical synapse strength by different Ca^2+^-dependent processes is highly analogous to the differences in synaptic activity that drive long-term potentiation and long-term depression in central glutamatergic synapses. The Cx36-GCaMP biosensor we have designed will be useful to investigate these differences.

## Synthesis

Reviewing Editor: Pablo Castillo, Albert Einstein College of Medicine Decisions are customarily a result of the Reviewing Editor and the peer reviewers coming together and discussing their recommendations until a consensus is reached. When revisions are invited, a fact-based synthesis statement explaining their decision and outlining what is needed to prepare a revision will be listed below. The following reviewer(s) agreed to reveal their identity: Georg Zoidl.

Your manuscript was evaluated by two experts in the field. Both reviewers agree on the relevance of your new tool to study calcium-dependent plasticity at electrical synapses, but they also raised concerns regarding the quantification and relatively low power (i.e. low “n’s”) of your results. In addition, there are a number of technical issues and clarification points that you should address. Below please find their specific comments. REVIEWER 1:

Connexin36 is the main neuronal connexin and forms electrical synapses in a variety of tissues, including the retina and olfactory bulb. Electrical synapses have long been regarded as rather static entities but recent evidence demonstrates that electrical synapses can undergo activity-dependent changes, which have been linked to local Ca2+ changes. To study these local Ca2+ changes at Cx36-containing synapses, the author generated a Cx36-GCaMP construct and demonstrate its suitability to study gap junction plasticity in a cell culture system. For this, the authors co-transfected cells with Cx36-GCaMP and various constructs, like NMDA receptor subunits and PSD95, to study potentiation of gap junctions in an in vitro system. They found that the Cx36-GCaMP construct represents a good tool to measure Ca2+ close to Cx36-containing gap junctions. Interestingly, they also found that HeLa cells do not require to be co-transfected with glutamate receptors to show glutamate receptor-driven potentiation, partially mediated by CaMKII.

This is an elegant study and the authors convincingly demonstrate that they generated an exciting new tool to study the plasticity at Cx36-containing gap junctions. However, there are some issues that need to be addressed:

It would be good to see some raw images of the tracer coupling experiments. Similarly, it would be of interest to see some fluorescent images of the NMDA receptor-transfected cells. Do the receptors occur in close proximity of the gap junction?

Figure 2 should be labeled in a way that it is self-explanatory. The authors should discuss why it appears as if the increase in fluorescence occurs even before the glutamate pulse at times.

The purpose of the experiment with PSD95 is not entirely clear. Maybe it would be good to add one or two sentences. Likewise it seems debatable whether PSD-95 and Cx36 are associated in photoreceptors. PSD-95 is situated in the membrane of the terminals but does it really coincide with Cx36?

The authors argue that the decline in fluorescence they see when applying ionomycin is due to Ca2+ extrusion mechanisms which act very fast. Would it be possible to demonstrate this directly, by using a blocker, e.g., for the ATPase involved?

The number of experiments/gap junctions/transfections is not always clear. It would be good to mention the number of independent experiments/transfections in addition to the number of gap junctions. Similarly, the n seems surprisingly low for some experiments (Figure 5-7).

REVIEWER 2

Authors (Required)

A novel Cx36-GCaMP3 fusion construct has been developed which effectively reports the changes to the Ca2+ microenvironment in the vicinity of Cx36 gap junctions. The authors claim that this tool will be valuable to investigate the dynamic changes in Ca2+ that are responsible for some forms of electrical synapse plasticity. They also provide information suggesting that Hela cells, a widely used model system for in vitro studies, express endogenous glutamate receptors that effectively drive intracellular calcium/calmodulin-dependent kinase II signaling. This cellular makeup can be exploited in many types of studies, most importantly to investigate bi-directional modulation of electrical synapse.

Overall this manuscript is well written with no major flaws and it is possible to agree on the claims made. The research shown has broad application potential beyond the focus of this manuscript. Technical limitations holding back an in-depth understanding of the mechanisms driving synaptic plasticity at electrical synapses can be overcome. Although research in this study was performed in vitro it will take one step to develop virus vector systems to express Cx36-GCaMP3 in vivo in a variety of models opening up for studies of Cx36 plasticity in a more natural context in combination with advanced imaging methods. Replacing GCaMP3 with alternative genetically encoded reporters, for example voltage sensors, could be the next step to investigate the dynamics of the microenvironment around GJPs.

Nevertheless, a number of minor to moderate issues must be addressed.

A potential weakness of this study is the choice of the cell model. Although Hela cells are widely used, distribution across the globe and long-term use have created diversity. In the future comparing results between different groups might be difficult. It would be helpful if the authors could establish a reference point for future comparison by reporting real-time PCR data (or protein data) for the expression of genes reported in this study. Test for endogenous Cx expression would be helpful.

It is unclear how the use of HEK cells is adding value to this study. The authors report that HeLa

cells displayed a more variable proportion of Cx36-GCaMP at cell-cell boundaries vs.

intracellular membrane compartments, making selection of regions of interest for quantification more difficult. Although this might be annoying it should not be a reason holding back efforts to collect the data required. Perhaps using Neuro2a cells which make small GJPs more comparable to Cx36 GJPs in vivo situation might have been a better choice. Neuro2a cells express both kainate and NMDA receptors. Consider showing GJPs between Hela cells to align with other data in the manuscript.

The Cx36-GCaMP3 fusion construct has been generated in a way that the Cx36 PDZ binding site is not accessible. In the literature this interaction domain is considered relevant for interaction with scaffolding proteins. This has been discussed, but have the authors tried to address this difference experimentally by extending the GCaMP3 reading frame and adding the relevant amino acids?

Statistics are a bit confusing. For example, it is unclear why either 2-way ANOVA with Sidak's multiple comparison tests (line 268) or 2-way ANOVA with Tukey's multiple comparison tests (line 282) were applied. Why using one or the other? In general terms, this manuscript would benefit from a statistics section. The data do not appear to be normally distributed. Are all requirements fulfilled for parametric tests, or are non-parametric tests appropriate? Provide details.

Is the analysis of 3-11 GJPs/cell pairs across different experiments sufficient? Data points are broadly distributed including potential outliers. Has statistical power been calculated to determine the probability of making a Type II error? Are all analyzed GJPs from one experiment? If three independent experiments were performed, how could it be that only 3 cell pairs were analyzed in some experiments.

From Figure 2A-C, initial baseline seems to be gradually declining. Could this be indicative of bleaching? Consider addressing bleaching as a factor in the data set.

Line 259: Unclear definition of "response area under the response curve."

Consider adding western blot data demonstrating expression of the proteins introduced into the cell model. Although an IRES based co-expression system was used it is unclear whether combinations of proteins are expressed equally. It is surprising that cells were already used 24hrs post transfection at a time before usually expression peaks. Any particular reason?

Line 16: use CaMKII consistently

Consider citing Front Mol Neurosci. 2019 Aug 28;12:206. doi: 10.3389/fnmol.2019.00206

## Author Response

We thank the reviewers for their thoughtful commentary on our manuscript. We have done some additional experiments and made a number of changes in the manuscript to improve its quality. We hope that these changes will answer the concerns of the reviewers. In the document below, I copy the questions or comments of each reviewer and describe how we have addressed them in the manuscript.

Reviewer 1.

1. This is an elegant study and the authors convincingly demonstrate that they generated an exciting new tool to study the plasticity at Cx36-containing gap junctions.

Thank you.

2. It would be good to see some raw images of the tracer coupling experiments. Similarly, it would be of interest to see some fluorescent images of the NMDA receptor-transfected cells. Do the receptors occur in close proximity of the gap junction?

We have added some images of tracer coupling, along with tracer fluorescent intensity and fits of the data with the compartmental diffusion model. We have also added a figure showing immunostaining of each of the transfected NMDA receptors and the Cx36-GCaMP. In general, transfected glutamate receptors cover most of the surface of the cell, so some receptors are in close proximity to the gap junctions, while a substantial population is at a distance.

3. The purpose of the experiment with PSD95 is not entirely clear. Maybe it would be good to add one or two sentences. Likewise, it seems debatable whether PSD-95 and Cx36 are associated in photoreceptors. PSD-95 is situated in the membrane of the terminals but does it really coincide with Cx36?

The inspiration for the PSD-95 experiments was, in fact, the close proximity of this scaffold with Cx36 reported in rod photoreceptor terminals (https://www.ncbi.nlm.nih.gov/pubmed/23407968) and the occurrence of NMDA receptors reported in the same location (https://www.ncbi.nlm.nih.gov/pubmed/10745222?dopt=Citation). We did not make this very clear in the manuscript, aside from simply citing these studies. The point is moot, however, as we have removed the PSD95 experiments from the manuscript. These experiments did not seem to fit with the other data, and with the additional experiments needed to address other critiques, the PSD95 data were not strictly comparable to the control data.

4. The authors argue that the decline in fluorescence they see when applying ionomycin is due to Ca2+ extrusion mechanisms which act very fast. Would it be possible to demonstrate this directly, by using a blocker, e.g., for the ATPase involved?

We have done some additional experiments, using available inhibitors for the Sodium-Calcium exchanger and the ER calcium ATPase. We did not find practical inhibitors for the plasma membrane calcium ATPase. In the new experiments, extrusion/sequestration of Ca2+ during ionomycin perfusion tended to bring Ca2+ to a plateau, but Ca2+ was rapidly reduced during washout. Blocking the Na-Ca exchanger blocked recovery, while blocking the ER calcium ATPase quickly elevated basal calcium and curiously dampened the ionomycin-induced rise in intracellular calcium. These new data have been added as a new figure and we have added some discussion of the topic.

5. The number of experiments/gap junctions/transfections is not always clear. It would be good to mention the number of independent experiments/transfections in addition to the number of gap junctions. Similarly, the n seems surprisingly low for some experiments (Figure 5-7).

We have added in numbers of transfections in the GCaMP imaging perfusion data in which n=number of gap junctions, and have performed some additional experiments for conditions/NMDA receptor combinations with low n. The other experiments with “low n” are tracer coupling experiments, all of which involve three separate transfection experiments, except in one case when one drug of a set didn’t work and a second set of three experiments was done with three of the six conditions. As already described in the methods, the underlying data involve 5-8 individual measurements of tracer coupling per condition per experiment, which we had averaged to yield one value for each experiment. We have debated in the past whether to report all of the individual measurements, and have discussed this with a biostatistician. The conclusion was that it would be acceptable to report all of the measurements, rather than just the mean of each experiment. We have done so in the revised manuscript, so the n’s are in the range of 18-30 and all measurement values are shown in the figures. We have also added a table of statistical tests and outcomes that lists both the number of measurements and the number of experiments performed for each comparison.

Reviewer 2

6. A potential weakness of this study is the choice of the cell model. Although Hela cells are widely used, distribution across the globe and long-term use have created diversity. In the future comparing results between different groups might be difficult. It would be helpful if the authors could establish a reference point for future comparison by reporting real-time PCR data (or protein data) for the expression of genes reported in this study. Test for endogenous Cx expression would be helpful.

We have always tracked passage numbers from the original HeLa CCL2 cells purchased from ATCC and have now reported these in the methods. In general, we have kept these passage numbers low so that the cell line we use is quite similar to the cell line widely available (ATCC does not know the passage number since the original derivation of HeLa). We had previously undertaken to identify the connexin background in HeLa cells through rtPCR and had planned to publish this as its own paper. In reviewing the size of the primer table and the data table, we have opted not to include these data in this manuscript so as to try to keep the focus on the characterization of the Cx36-GCaMP biosensor. However, we have also examined RNASeq data from HeLa cells deposited in GEO (Gene Expression Omnibus), confirming expression of several connexin genes in HeLa cells and confirming expression of NMDA-type glutamate receptors. We have included these data in a new table.

7. It is unclear how the use of HEK cells is adding value to this study. The authors report that HeLa cells displayed a more variable proportion of Cx36-GCaMP at cell-cell boundaries vs.

intracellular membrane compartments, making selection of regions of interest for quantification more difficult. Although this might be annoying it should not be a reason holding back efforts to collect the data required. Perhaps using Neuro2a cells which make small GJPs more comparable to Cx36 GJPs in vivo situation might have been a better choice. Neuro2a cells express both kainate and NMDA receptors. Consider showing GJPs between Hela cells to align with other data in the manuscript.

We understand the reviewer’s opinions, but for the purpose of this study, which was to validate the new biosensor, the HEK cell expression system was by far the most suitable. As it is, the experiments reported constituted more than two years effort for the lead author of the manuscript. It would be highly impractical to repeat all of that in another cell line, especially if the experiments are more difficult to perform and the results would not be different.

8. The Cx36-GCaMP3 fusion construct has been generated in a way that the Cx36 PDZ binding site is not accessible. In the literature this interaction domain is considered relevant for interaction with scaffolding proteins. This has been discussed, but have the authors tried to address this difference experimentally by extending the GCaMP3 reading frame and adding the relevant amino acids?

The GCaMP biosensors work by having calmodulin at the C-terminus of the sensor reach back and bind to a calmodulin target sequence at the N-terminus of the sensor, bringing the two halves of the circularly permuted GFP closer together so that fluorescence is increased. If one were to add the PDZ binding motif of Cx36 to the C-terminus of the GCaMP, this would hinder the movement of the C-terminal portion and would likely prevent its fluorescence response to calcium. Thus, it is not advisable to add interaction motifs to the C-terminus of the biosensor. This is also the reason that this sensor cannot be embedded in the middle of a protein.

9. Statistics are a bit confusing. For example, it is unclear why either 2-way ANOVA with Sidak’s multiple comparison tests (line 268) or 2-way ANOVA with Tukey’s multiple comparison tests (line 282) were applied. Why using one or the other? In general terms, this manuscript would benefit from a statistics section. The data do not appear to be normally distributed. Are all requirements fulfilled for parametric tests, or are non-parametric tests appropriate? Provide details.

Sidak’s or Dunnett’s multiple comparisons are used when specific comparisons are planned, e.g. each condition to a single control condition. One may be preferred over the other in certain experimental designs, but they are similar. We have now eliminated experiments in which Sidak’s multiple comparisons are most appropriate, so these are no longer in the manuscript. Tukey’s multiple comparisons are used when each condition is compared to every other condition. In practice, for all of our experiments in which we use Tukey’s multiple comparisons, only specific comparisons are relevant (and have been pre-planned), such as glutamate exposure vs. glutamate + PKA inhibitor, glutamate vs. glutamate + CaMKII inhibitor, and control vs. all three treatments. In practice, the tests can't account for that many specific combinations, so the more general comparison of every condition to every other is used, and certain irrelevant comparisons are ignored. We have added a statistics section to the methods as suggested, and added a statistical results table.

10. From Figure 2A-C, initial baseline seems to be gradually declining. Could this be indicative of bleaching? Consider addressing bleaching as a factor in the data set.

This is what we believe to cause the declining baseline. This was already stated in the methods along with our description of the mathematical methods to remove the baseline decline from the data (lines 164-166 of the original manuscript). We generally fit a first order exponential to the baseline, but in some cases needed to use a linear baseline if there was no decline or if a first-order exponential was a poor fit.

11. Line 259: Unclear definition of “response area under the response curve.”

This is the integrated area under the response (ΔF/Fo*sec). This is basically the sum of the ΔF/Fo measurements for each point above baseline in the response, normalized to 1 second time intervals. We have added text to the methods to clarify this.

12. Consider adding western blot data demonstrating expression of the proteins introduced into the cell model. Although an IRES based co-expression system was used it is unclear whether combinations of proteins are expressed equally. It is surprising that cells were already used 24hrs post transfection at a time before usually expression peaks. Any particular reason?

We have added immunofluorescence labeling, as suggested by the other reviewer. The dual expression vector we used actually has two separate promoters for the two genes expressed (EF1a for Cx36-GCaMP and CMV for NR1). The IRES element was added after the Cx36-GCaMP to attempt to express the puromycin resistance gene for generating stable cell lines. As the reviewer warns, this was not particularly effective, and we were not able to generate stable cell lines with that strategy. The cells were used 24 hours after transfection because NMDA receptors transfected into HEK cells kill the cells. The 24-hour time point is a compromise in which we still have enough living cells to work with. Cx36-GCaMP expression level and gap junctions were rarely a problem. Indeed, there were often many cells expressing the proteins at such a high level that Cx36-GCaMP expression was delocalized all over the surface of the cell. These could not be used for experiments, but our recordings show that these cells displayed very large calcium responses, no doubt because the NMDA receptor expression was very high and there was a large area of Cx36-GCaMP available to respond to changes in cytoplasmic calcium.

13. Line 16: use CaMKII consistently.

This is in the abstract, where it is necessary to spell out the protein name. Unfortunately, due to the abstract word limit, I shortened it in a non-standard way. I have modified the abstract in other ways to spell this out completely now.

14. Consider citing Front Mol Neurosci. 2019 Aug 28;12:206. doi: 10.3389/fnmol.2019.00206

Thank you for pointing this out. We had cited an earlier paper from this group describing the co-localization of CaMKII with Cx36 in the retina, but this paper also describes this association. We have added it in.
